# Fish Disease Occurrence and Immune Prevention and Control

**DOI:** 10.3390/vaccines14090747

**Published:** 2026-08-28

**Authors:** Yonghua Hu

**Affiliations:** Institute of Tropical Bioscience and Biotechnology, Chinese Academy of Tropical Agricultural Sciences, Haikou 571101, China; huyonghua@catasitbb.cn

Aquaculture has rapidly emerged as one of the fastest-growing industries, supplying nearly half of all aquatic food products intended for human consumption. As production intensifies, however, disease outbreaks have become an enduring challenge, causing severe economic losses and threatening the sustainability of the sector. Vaccination stands as the most efficient and environmentally friendly tool for disease prevention in aquaculture, yet the development of effective vaccines requires a thorough understanding of host–pathogen interactions, fish immunity, and the mechanisms by which vaccines elicit protective responses.

This Special Issue, “Fish Disease Occurrence and Immune Prevention and Control”, was launched to collect cutting-edge research on these topics. The five papers published in this issue—comprising four original research articles and one comprehensive review—collectively advance our understanding of fish disease pathogenesis, vaccine development, and immune prevention strategies. Together, they address key challenges in aquaculture vaccinology, from bacterial pathogenesis and vaccine efficacy evaluation to innovative vaccine delivery technologies.

## 1. Pathogenesis of Aeromonas Infections

Two papers in this Special Issue focus on the pathogenic mechanisms of *Aeromonas* species, which are among the most significant bacterial pathogens in aquaculture. Wang et al. investigated *Aeromonas veronii* infection in crucian carp, demonstrating that vaccination with an inactivated vaccine achieved a relative percent survival (RPS) of 73.33%. Through comprehensive analysis of immune-related gene expression, liver transcriptomics, and gill microbiota, the authors revealed that BAFF transcription across organs emerged as a robust sentinel readout of vaccine-elicited immune activation. Notably, the study identified marked immune–metabolic coupling in the liver following challenge and suggested coordinated immunophysiological interplay between the liver and the spleen. The gill microbiota of vaccinated fish remained broadly closer to that of the control group in overall composition, indicating that vaccination helped maintain mucosal ecological homeostasis.

Tang et al. explored a different dimension of *Aeromonas* pathogenesis by investigating amonabactin synthetase G (AmoG) in *A. hydrophila*. The authors demonstrated that the deletion of *AmoG* impaired the bacterium’s iron-chelating ability and reduced its pathogenicity. Mechanistically, AmoG was found to modulate host Wnt/β-catenin signaling and gut mucosal barrier function. Infection with the Δ*AmoG* mutant resulted in increased goblet cells, enhanced expression of tight junctions, and lower levels of D-lactic acid and lipopolysaccharide (LPS), indicating improved gut barrier integrity. This study provides novel insights into how *A. hydrophila* exploits nutrient acquisition mechanisms to disrupt host defenses, offering potential therapeutic targets for disease control.

## 2. Vaccine Development and Delivery Innovations

Two original research articles in this issue evaluate the efficacy of different vaccine approaches under laboratory and field conditions. Mohd Ali et al. assessed a feed-based genome-free bacterial vaccine against *A. hydrophila* in red tilapia, employing SimCells^®^ technology to incorporate genome-free bacterial cells into feed. The vaccine, delivered orally with a booster at week 2, significantly improved RPS following bacterial challenge. Vaccinated fish showed upregulated expression of immune-related genes (IL-1β, MHC-II, CD4, IgT, and IgM) in the hindgut and head kidney, as well as enhanced serum lysozyme activity and overall IgM production in serum, skin mucus, and gut lavage. This study demonstrates that genome-free bacterial vaccines delivered via feed represent a promising and practical approach for controlling motile *Aeromonas* septicemia in cultured tilapia.

Min et al. evaluated a formalin-inactivated vaccine against red seabream iridovirus (RSIV) in rock bream under both laboratory and field conditions—a critical step often overlooked in vaccine research. In a large-scale field trial involving over 100,000 fish from two commercial marine cage farms in Korea, the vaccine demonstrated an RPS of 54.67% under natural infection conditions. Cumulative mortality was 36.43% in the vaccinated group compared to 80.32% in the control group. Neutralizing antibody titers mirrored the mortality results, confirming that the vaccine elicited protective humoral immunity. This study provides compelling evidence that inactivated vaccines can be effective against viral pathogens in real-world aquaculture settings, bridging the gap between laboratory efficacy and practical applicability.

## 3. A Comprehensive Review of Aquaculture Vaccinology

Tammas et al. contributed a comprehensive review that contextualizes the advances presented in this Special Issue within the broader landscape of aquaculture vaccinology. The review assesses recent developments and milestones in the field, covering emerging vaccine technologies, innovative delivery methods, novel adjuvants, and parasite vaccine development. The authors highlight how breakthroughs in nanotechnology, biotechnology, and artificial intelligence—ushered in by the -omics era—are driving increased species-specific precision and improved vaccine-delivery systems. The review also provides a strengths, weaknesses, opportunities, and threats (SWOT) analysis of the sector, offering a valuable resource for researchers and industry stakeholders alike.

## 4. Conclusions and Future Perspectives

The papers collected in this Special Issue reflect the current state and future directions of fish disease prevention and control. Several key themes emerge. First, understanding the molecular mechanisms of pathogen virulence—whether through siderophore-mediated iron acquisition or the modulation of host signaling pathways—is essential for identifying targets for vaccine development and therapeutic intervention. Second, vaccine efficacy must be evaluated not only through survival metrics but also through comprehensive immune profiling, including gene expression analysis, antibody responses, and mucosal immunity. Third, innovative vaccine delivery systems, such as feed-based oral vaccines, offer practical advantages for large-scale aquaculture operations. Fourth, field validation of vaccine efficacy is critical to ensure that laboratory findings translate to real-world protection.

Looking ahead, the integration of multi-omics approaches, the development of species-specific vaccines, and the refinement of delivery methods will continue to drive progress in aquaculture vaccinology. As the review by Tammas et al. emphasizes, a unified perspective that bridges fundamental immunology and practical application is essential for addressing the persistent health challenges facing the aquaculture industry. We hope that the contributions in this Special Issue will inspire further research and innovation, ultimately contributing to more sustainable and resilient aquaculture production worldwide.

